# Advantages of Robotic Surgery for Patients of Reproductive Age with Endometrial Cancer

**DOI:** 10.3390/life14091108

**Published:** 2024-09-03

**Authors:** Magdalena Bizoń, Zuzanna Roszkowska, Renata Kalisz, Łukasz Szarpak, Maciej Olszewski

**Affiliations:** 1 LUX MED Oncology Hospital, św. Wincentego 103, 03-291 Warsaw, Poland; magdalena.bizon1@gmail.com; 2 Students’ Scientific Club “ROBOTICS”, Medical University of Warsaw, 02-091 Warsaw, Poland; s082681@student.wum.edu.pl (Z.R.); renata.kalisz5@gmail.com (R.K.); 3 Department of Clinical Research and Development, LUXMED Group, 02-676 Warsaw, Poland; lukasz.szarpak@luxmed.pl; 4 Henry JN Taub Department of Emergency Medicine, Baylor College of Medicine, Houston, TX 77030, USA

**Keywords:** endometrial cancer, minimally invasive surgery, robot-assisted laparoscopy, young age, BRCA mutation

## Abstract

This review presents current knowledge on the surgical treatment of endometrial cancer in young patients. Endometrial cancer is the most common gynecological cancer in Europe. Higher morbidity is correlated with obesity, hypertension and diabetes, which are growing worldwide. However, endometrial cancer at an early age is very rare. The first line of treatment for this cancer is radical hysterectomy, which is controversial in young women. There is an alternative method of fertility-sparing treatment. However, there is a group of young patients for whom surgical treatment is recommended. According to European guidelines, minimally invasive surgery is recommended for endometrial cancer. The aim of the study was to present the advantages of robotic surgery for endometrial cancer detected at a young age. The procedure of radical treatment with robot-assisted laparoscopy is more precise. Better visualization and stabilization of instruments allow a shorter procedure time, a brief hospital stay and fewer complications. Quality of life may be at a similar level. Incisions after trocars are painless and more esthetic than a classical wound. Bilateral adnexectomy in endometrial cancer depends on age, molecular status of the cancer, stage, genetic risk factors and individual decision. Conclusions: Robotic surgery seems to be a better surgical method for endometrial cancer in younger patients.

## 1. Introduction

Endometrial cancer is the fourth most common malignant disease among women, representing 7% of all new cancers, with over 400,000 new cases per year worldwide. Endometrial cancer is usually detected in postmenopausal women. Statistics show that the percentage of new cases of endometrial cancer is 16.7% in women between the ages of 45 and 54, 34.5% in those aged 55–64 and 25.8% in those aged 65–74 [1]. The risk of cancer in women under the age of 39 is low, at 5%. A higher rate of cancer in women at this age is observed for ovarian cancer (12%) and a lower rate for cervical cancer (2%) [2].

At a young age, racial disparities are clearly apparent. Aggressive endometrial cancer at an advanced stage is diagnosed in black women more frequently than in white women below 40 years old [3].

Endometrial cancer is often diagnosed in developed countries. It is correlated with the longer-lasting influence of estrogen. A high level of this hormone is present among women with menarche at a younger age, women at a late age of menopause, those with nulliparity and those having the first child at a late age [4]. Adipose tissue present in obesity is a source of estrogen. The level of endogenous estrogen is higher, and sex hormone-binding globulin (SHBG) decreases. This hormonal imbalance causes the proliferation of endometrial epithelial cells and increases the risk of oncopathogenesis [5].

Currently, according to the European Society of Gynecological Oncology (ESGO) guidelines, four main types are included: POLE-ultramutated endometrial cancer (EC), MMR-deficient/microsatellite instability (MSI) EC, P53-mutated EC and non-specific molecular profile (NSMP) EC.

Molecular identification is performed on endometrial tissue. Each molecular type is associated with a different risk group, allowing specific adjuvant treatment to be provided. The current FIGO classification contains not only the staging of endometrial cancer but also the molecular type of the disease [6,7].

Minimally invasive surgery has played a role in surgical procedures for 30 years and is recommended in endometrial cancer according to the ESGO/European Society for Radiotherapy and Oncology (ESTRO) and European Society for Medical Oncology (ESMO) guidelines [6,8].

Currently, minimally invasive surgery is one of the surgical methods in gynecological oncology. If fertility-sparing treatment is not possible, performing a hysterectomy at a young age can induce menopausal symptoms. The selection of the best surgical method makes it possible to improve the quality of life after endometrial cancer. The population of patients with endometrial cancer at a young age can have a different point of view on the type of surgery than peri- and postmenopausal women.

The aim of the study was to indicate the benefits of robotic surgery for patients with endometrial cancer diagnosed at a young age.

## 2. Materials and Methods

The review is based on papers from the medical databases PubMed, Cochrane Library, Web of Science and Scopus, which were chosen according to selective criteria. The aim of the research was to identify the advantages and disadvantages of robotic surgery for young women with endometrial cancer.

We included 2526 articles, reviews, meta-analyses, editorials and letters in English about robotic surgery in endometrial cancer of patients of reproductive age for further analysis. We excluded papers in languages other than English.

Search terms: endometrial cancer in reproductive age; endometrial cancer in young age; robotic surgery; fertility sparing treatment; sexual life; endometrial cancer survivors; quality of life; robotic surgery in young age.

Time frame/years searched: between 2010 and 2024.

## 3. Discussion

### 3.1. Epidemiology of Endometrial Cancer at Reproductive Age

Five per cent of cases of endometrial cancer are diagnosed in patients under the age of 39. In a retrospective study of a tertiary care center in Michigan from 2006 to 2017, only three cases of endometrial cancer and five cases of complex atypical endometrial hyperplasia were registered [9]. Otherwise, statistics for 2021 in Poland revealed only three cases per year of endometrial cancer below the age of 25. These data confirm very rare cases. Because of the early stage of the disease and young age of the patients, the target is to maintain fertility. An MRI of the pelvis is performed to detect infiltration of endometrial tissue and to choose the conservative or surgical way of treatment [2,10,11]. Most cases of endometrial cancer at a young age are associated with obesity (BMI > 30), smoking and polycystic ovarian syndrome (PCOS) [9].

Endometrial cancer at a young age is usually detected during hysteroscopy because of abnormal bleeding or due to the fertility diagnostic process [12].

Usually in women under 40 years old, a characteristic feature of endometrial cancer is a highly differentiated focal endometrioid tumor with minimal invasion of the myometrium. High expression of estrogens and progesterone receptors is specific in these cases [13]. Assessment of myometrial and cervical stromal invasion are predictive factors of the stage of endometrial cancer. Savelli et al. confirmed good accuracy in local staging of endometrial cancer using transvaginal ultrasound, while magnetic resonance, because of its expensive costs, should be dedicated only in cases of poor quality ultrasounds [14]. However, in order to perform a lymphadenectomy, frozen section intraoperative seems better than preoperative transvaginal ultrasound [15]. In comparison to transvaginal ultrasound, two-dimensional volume contrast imaging has higher accuracy and reliability than three-dimensional imaging [16].

**Figure 1:**
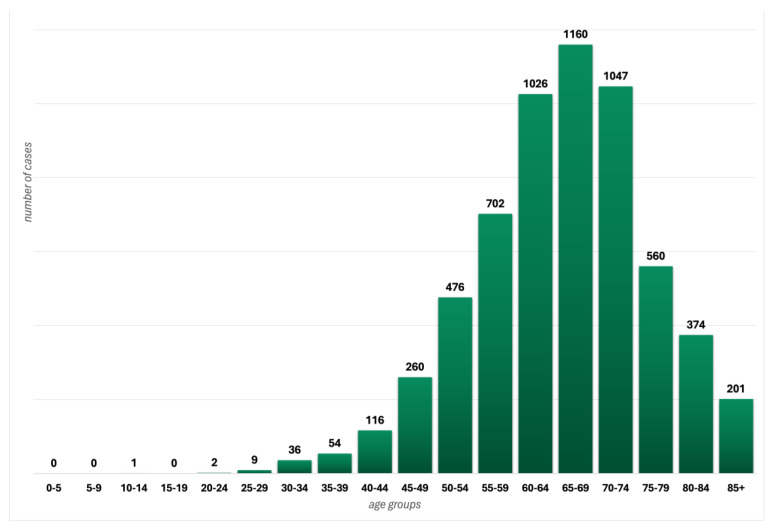
Morbidity of endometrial cancer in 5-year age groups in Poland in 2021.

The epidemiology of EC in Poland is presented in Figure 1.

### 3.2. Risk Factors of Endometrial Cancer at a Young Age

The most common risk factor is obesity, which appears after the age of 20. It is associated with less physical activity, higher insulin resistance, hypertension and diabetes type II. Longer exposure to estrogens, such as in women with early menarche or anovulatory menstrual cycles during PCOS, increases the morbidity of endometrial cancer. On the other hand, there is a genetic background correlated with genetic mutations or Lynch syndrome [13].

Some of the risk factors for endometrial cancer, such as obesity and nulliparity, can influence the risk of complications during pregnancy. On the other hand, smoking, anovulation and endometriosis increase the risk of infertility. A Cochrane review concluded that a cumulative dose of clomiphene citrate above 2000 mg used to stimulate ovulation might increase the risk of endometrial cancer. Some studies have noted a higher risk of endometrial cancer in women undergoing ART. Nevertheless, some meta-analyses revealed no risk of oncogenesis in infertile patients. However, the similar factors of infertility and endometrial cancer can be a reason for oncogenesis, not only assisted reproduction techniques [17]. To sum up, considering the higher risk of endometrial cancer in women at a younger age, it is very important to be prepared for the best method of treatment in case of the development of this cancer [18].

However, some behaviors can reduce the morbidity of endometrial cancer. A meta-analysis by Hidayat et al. demonstrated that physical activity reduces the risk of this disease [19]. Additionally, measurement of blood pressure, diet and control of diabetes and hypertension protect against uterine cancer [6]. A good solution is an active lifestyle and the avoidance of sedentary habits [17]. Research shows that oral contraception (OC) taken for 1 year reduces the risk of endometrial cancer by 20%. It has been observed that long-lasting usage of OC for more than 10 years reduces the risk of oncogenesis by 80%. Oral contraception has an observed influence on lower morbidity in type 1 endometrial cancer [3,20].

### 3.3. Endometrial Cancer with Genetic Mutation Background

Endometrial cancer can be related to mutations in the *TP53* gene and the suppression of estrogen and progesterone receptors. Kovalenko et al. observed an association between the influence of methylated PTENP1 on expression of the *PTEN* gene and the prevention of endometrial cancer [21,22,23]. Levine et al. reported only 1.5% frequency of coincidence of endometrial cancer and *BRCA* mutation in a population of Ashkenazi Jewish women. No papillary serous endometrial carcinoma was diagnosed in patients with a genetic background. These cases were not etiologically similar to papillary serous ovary or fallopian tube cancer [24,25]. However, the presence of the *BRCA* mutation is a risk factor for breast and ovarian cancer [26,27]. If the *BRCA* mutation is confirmed, bilateral salpingo-oophorectomy should be considered at reproductive age.

A special risk group of patients consists of women with germline mutations of one of the MMR genes with a higher risk of Lynch syndrome [28,29]. The cumulative incidence of cancer increases with the age of the patients. At 70 years, the risk of endometrial cancer was found to be 51% and 41% for MSH2 and MSH6, and 34% and 24% for MSH1 and PMS2. This is the reason why risk-reducing hysterectomy and bilateral salpingo-oophorectomy should be performed before age 40 [30].

### 3.4. Fertility-Sparing Treatment at a Young Age

Fertility-sparing treatment is an option for women with a strong desire for pregnancy. It can be considered only in indications of atypical endometrial hyperplasia and endometrioid endometrial cancer in stage IA with histological stage G1 or G2, without myometrial invasion and confirmed molecular status of endometrial cancer without a familial genetic background [21,22,23,24,25,26,27,28,29,30,31,32,33,34,35,36,37,38,39,40].

Treatment is based on medroxyprogesterone acetate (MPA) or megestrol acetate (MA) [29]. However, during treatment, every 2–3 months, histological assessment is obligatory for monitoring the effects of this conservative therapy [41]. Oral progestin therapy alone is linked to a higher risk of recurrence and more systemic adverse effects [42,43]. Another method of fertility-sparing treatment to avoid systemic effects after oral therapy is a levonorgestrel-releasing intrauterine system (LNG-IUS), well tolerated in obese patients. Additionally, metformin and weight loss are recommended.

Combined use of oral medroxyprogesterone acetate and a levonorgestrel intrauterine system is also possible [44].

Nevertheless, fertility-sparing treatment is not always effective. A meta-analysis revealed a 12-month remission rate of 78% and a 32% rate of successful pregnancy. The 12-month recurrence rate was 9.6% [45].

### 3.5. History of Robotic Surgery

Robotic surgery is currently the newest technology for surgical procedures. For 30 years, it has been used in different branches of medicine, such as surgery, gynecology, urology, laryngology and others. In 2009, a study on the use of robotic surgery in gynecologic oncology was published for the first time. The number of robotic surgical systems increases every year. From 2007, over 6 years, the number of platforms doubled in Europe and the USA. In 2013, about 1.5 million robotic procedures were registered [46]. Currently, gynecological procedures are increasingly performed with the assistance of a da Vinci robot [47]. In 2024, in Poland, 43 robotic systems were registered.

### 3.6. Robotic Surgery in Gynecology

Robotic surgery is useful in gynecological oncology as well as in benign diseases. Thanks to a comfortable condition and a shorter learning curve, surgeons prefer this procedure to hysterectomy, myomectomy and sacrocolpopexy [48]. Nevertheless, good results and the most frequent use of robot-assisted laparoscopy make the robot suitable for oncological disorders such as endometrial and cervical cancer [49]. Robotic surgery can also be used for the restaging of ovarian cancer [50].

In benign gynecological disease, there are still a lot of indications for minimally invasive surgery assisted by a robot. This is the best method for endometriosis: to remove all endometrial implants and adhesions. Thanks to good visualization, it is possible to see many structures enlarged, to avoid damage to the adnexa and to improve the postoperative quality of life. Bowel resection and removal of foci of deep-infiltrating endometriosis can affect the infertility results. On the other hand, robotic surgery as a minimally invasive method is recommended in prolapse surgery. One of the most commonly used procedures is sacrocolpopexy. However, the costs of surgery are higher when robot-assisted laparoscopy is applied.

Moreover, there was no difference in long-term outcomes or shorter postoperative pain in robotic surgery in gynecology [51].

### 3.7. Robotic Surgery in Endometrial Cancer

Minimally invasive surgery is a recommended approach in endometrial cancer according to ESGO/ESTRO/ESP guidelines [6].

The procedure of surgical treatment of endometrial cancer at postmenopausal age consists of hysterectomy with bilateral salpingoophorectomy. However, in the case of women of reproductive age, performing an ovariectomy can induce menopausal symptoms.

Gu et al. revealed differences in overall survival in patients after bilateral salpingo-oophorectomy and only salpingectomy in the early stage of endometrial cancer and confirmed ovarian preservation as safe and can be considered in this stage of disease [52]. However, it is recommended to perform salpingectomy during hysterectomy in case of the risk of high-grade serous ovarian cancer [53].

On the other hand, there is a risk group of patients with BRCA mutation, Lynch syndrome and cancer family history for whom ovarian preservation is not recommended [54].

Minimally invasive surgery seems to be an indication at reproductive age.

**Figure 2:**
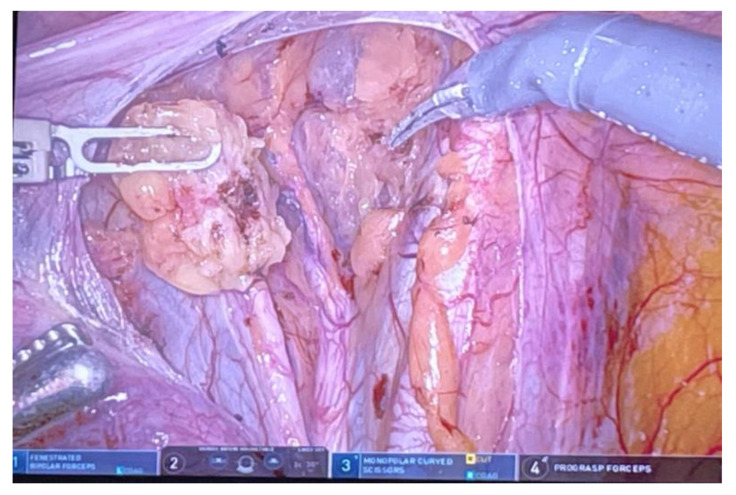
Sentinel lymph node.

**Figure 3:**
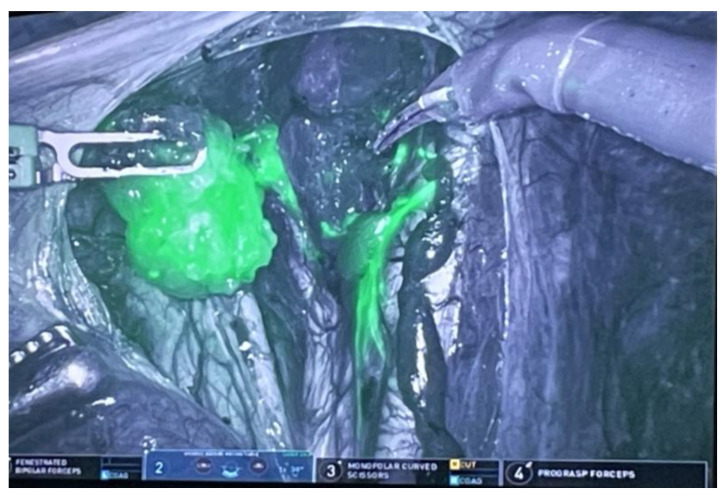
Sentinel lymph node detected using the FireFly function during robotic surgery.

Better visualization and precise movements during the procedure reduce blood loss and favor a quicker recovery. According to the ESGO recommendations, a biopsy of sentinel lymph nodes with subsequent ultrastaging avoids complications that can occur after the completion of a pelvic lymphadenectomy [55]. In robotic surgery, indocyanine green is used for indicated sentinel lymph nodes, and the FireFly function visualizes targeted lymph nodes during the procedure (Figure 2 and Figure 3).

Nevertheless, if advanced endometrial cancer is suspected, enlarged lymph node radical surgery is appropriate [56]. Minimally invasive surgery allows for quicker recovery and a faster onset of adjuvant treatment, which is extremely important at a young age. Adjuvant treatment should begin as soon as possible, because regional recurrence occurs in 4–20% of patients within the first two years after treatment [57].

Kim et al. compared the oncologic outcomes of 138 advanced-stage endometrial cancer patients, including recurrences, disease-free survival and overall survival. They observed a higher overall recurrence rate in the open surgery group than in the minimally invasive surgery group [58].

The study, which included over 1000 patients who underwent robotic surgery, showed that they were less likely to develop postoperative complications. The research compared 5 years of follow-up. Moreover, minimally invasive robotic surgery was also found to be more cost efficient [59]. Bruno et al. revealed a study that presented a risk assessment model for complications in minimal invasive surgery, including type of surgical technique, surgeon’s experience, body mass index and previous surgery [60].

**Figure 4:**
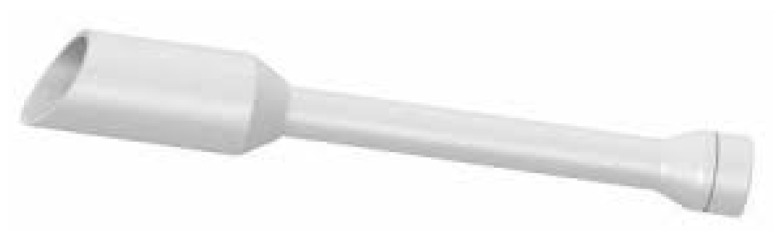
MyTube as a colpotomizer.

Saini et al. found a correlation between intra-operative tumor spillage and cancer recurrence. Use of an intrauterine manipulator increases the risk of recurrence due to specimen fragmentation of tissue into the peritoneum [61]. On the other hand, the use of a colpotomizer or endostapler prevents tumor spillage and seems to be a better solution in endometrial cancer for the prevention of recurrence [62]. Figure 4 presents one of the types of colpotomizer.

Surgery assisted by a robot can be performed using different systems. Meren et al. conducted a study comparing robotic surgery using multiport and single-site systems. The latter is used only in a few centers. Interestingly, no differences were found between these kinds of robotic surgery in terms of BMI and sentinel lymph node detection. There were no postoperative complications, and there were no differences in cosmetic results or quality of life. The only advantage detected was the shorter time of surgery using single-site robotic surgery [63].

### 3.8. Benefits of Robotic Surgery at a Young Age

Advantages of robotic surgery, such as 3-dimensial vision, an enlarged view and wristed instruments, are the benefits of precision movements, preparing tissue and avoiding destroying vessels or nerves. What is more, stable robotic arms avoid the tremor of human arms, which occurs in laparoscopy and the open approach [64,65]. Another advantage of robotic surgery is the magnification of the camera view, which is larger than in laproscopy [66].

However, there are some benefits of robotic surgery that are especially significant for younger patients.

Robotic instruments due to the wrist-like rotation have the possibility of precision movements, which are important in a robotic nerve-sparing radical hysterectomy [67,68,69]. These procedures are commonly performed in early-stage cervical cancer IA2 to IB1 as a hysterectomy type C1 according to the Querleu-Morrow classification. Puntambeka et al. conducted a case study of robotic nerve-sparing radical hysterectomy in 12 cases. No complications or local recurrences were registered. All patients were sexually active after the surgery. These results highlight the benefits of a robotic approach and oncological safety for these procedures in the early stages of cervical cancer [70].

Paek et al. compared robotic radical hysterectomy types C1 and C2 and revealed early bladder function return and feasible outcomes for C1 and early discharge [71].

Nerve-sparing surgery is also performed in deep infiltrating endometriosis, which showed an alternative way of treatment for pain relief in severe endometriosis [72].

Another important factor to consider is the learning curve of the robotic approach. Nerve-sparing surgery is a difficult surgical technique for beginner gynecologists with less experience. The robotic approach with an enlarged view allows visualization of all steps of procedures for the education of younger trainee assistants and seems to facilitate starting to perform nerve-sparing surgery earlier [73].

In most cases of endometrial cancer, there is no indication of nerve-sparing surgery. However, the ability to accurately prepare pelvic structures helps reduce the risk of complications during robotic procedures in every case and allows one to deal with unexpected circumstances in the event of complications, which is not so clear in other surgical techniques.

Sentinel lymph node biopsy as an element of surgical treatment of endometrial cancer improved patients’ overall quality of life and well-being in other parts of life [74]. Systematic pelvic lymphadenectomy is associated with a high risk of intraoperative complications during the preparation of vessels and nerves. Thanks to the enlarged view, stable 3D visualization, and the sentinel lymph node procedure, the risk of complications decreases.

What is more, an interesting RASHEC trial compared laparotomy and robot-assisted surgery. Patients who underwent surgical procedures had to assess their quality of life before and 12 months after the operation. All women diagnosed with high-risk endometrial cancer answered questions in a special questionnaire. Respondents reported a similar feeling about global health status. Their emotional and cognitive functioning were the same after one year of hospitalization. Cerebral hypoxia, which can lead to disorders in cognitive functioning, might be associated with the Trendelenburg position and postoperative chemotherapy. Symptoms assessed 12 months after the operation, such as fatigue, nausea and vomiting, pain, insomnia, appetite loss and diarrhea, were comparable. On the other hand, robot-assisted surgery allows for better sexual interest and sexual activity compared to laparotomy. Definitely lower percentages of lymphedema, muscular pain, urological symptoms and gastrointestinal symptoms were observed in patients 12 months after robot-assisted surgery. Additionally, fewer patients complain of tingling, hair loss and taste changes one year after laparotomy [75,76,77]. All these benefits are extremely important for younger patients to maintain quality of life after surgery.

Another factor favoring a more precise technique is the fact that the percentage of younger patients with endometrial cancer and endometriosis is higher, which results in more difficulties in the abdominal cavity during surgery [78]. Adhesion removal and preparation of tissue in an avascular area are possible thanks to wrist instruments, better visualization and an enlarged view. What is more, robotic surgery is more precise than the laparoscopic approach, as was confirmed in the observational study reported by Sinha et al., which showed less follicular loss area after cystectomy performed by the robotic method than laparoscopy [79].

Another benefit of robot-assisted laparoscopy is a lower level of pain. Wright et al. noted more commonly persistent opioid use among younger women and patients with depression, anxiety and substance use disorders [80]. Currently, an increasing frequency of depression is reported, especially in young people. When compared with the level of pain after minimally invasive surgery, there was no need for continued opioid use after hospitalization. Benefits of robotic-assisted laparoscopic hysterectomy compared to conventional laparoscopy and vaginal hysterectomy include reduced blood loss and postoperative pain [51,81,82].

From a psychological perspective, cancer at reproductive age has an impact on the further life of the patient. A lack of possibility of fertility-sparing surgery can influence the relationship with the partner. The need for adjuvant therapy can lead to prolonged rest without work, which can strain financial budgets and impair social relationships.

So et al., in 2022, assessed 1016 patients after a 6-month cancer treatment. In this group, 72 patients had gynecological cancers. Younger patients returned to work significantly, sooner and the risk of depression was lower than for the patients on sick leave or unemployed [83].

Robotic surgery, with a shorter recovery than other techniques, allows for a quicker return to work and to society, leading to less stigmatization of such persons with neoplastic disease [83].

Sexual life is extremely important and has a significant impact on endometrial cancer survivors [84].

Hysterectomy performed at reproductive age can lead to low libido and a lack of sexual interest. Moreover, it is frequently correlated with vaginal dryness, pain during intercourse and a shorter vagina after the procedure [85]. The use of a colpotomizer reduces the risk of a larger vaginal cuff and allows for better orgasms during intercourse. Patients using a dilatator and lubricants after treatment of endometrial cancer reported better quality of sexual life [86,87]. No study has focused on sexual quality of life only after robotic surgery; however, Datta et al. reported that sexual activity and functions were at a higher level after surgical treatment only than in groups after surgery with adjuvant vaginal brachytherapy and surgery followed by chemotherapy and radiation [88].

Sobocan et al. published a study that investigated quality of life after treatment of endometrial cancer. Out of the whole group, 66.2% underwent minimally invasive surgery. In sexually active women from the whole group, sexual and vaginal problems were associated with poor body image and muscular pain. An advantage of robotic surgery is the impact on body image and reduction in anxiety associated with this area. Muscular pain is stronger after open surgery when damage to muscles is more severe, and wound healing lasts longer than after robotic surgery [89].

Additionally, the importance of sexual quality of life was described by Roussin et al. in their study, which showed the vital role of healthcare providers in protecting and improving sexual quality of life after gynecological cancer [90].

**Figure 5:**
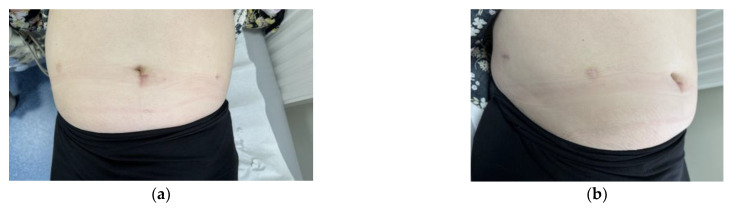
Scars of a 39-year-old patient with endometrial cancer after robotic hysterectomy (**a**) in frontal view; (**b**) in lateral view.

Finally, minimally invasive surgery (MIS) is an innovation in healthcare technology. The evolution of surgical methods allows patients to recuperate faster with a shorter hospital stay. A wound after laparotomy is bigger and causes trauma for patients. MIS reduces this wound access trauma and causes fewer postoperative complications. There is no problem with hernia because the trocar points leave only small wounds. This reduces the problem of hernia and disfigurement of the skin [91]. These advantages are significant, especially for young women who do not want to have many scars [92]. Scars after robotic surgery are presented in Figure 5a,b.

However, the disadvantages of robotic surgery are the costs of the procedure, which are more expensive than laparoscopy. Yoon et al. compared robotic and laparoscopic surgery in endometrial cancer and observed a significantly higher rate of total costs in the case of robotic surgery with no differences in oncological outcomes [93].

## 4. Conclusions

Robotic surgery seems to have more benefits for patients of reproductive age with endometrial cancer. In these cases, a high-risk genetic background is a contraindication for fertility-sparing treatment. The benefits of using this surgical technique are still increasing. The young age of a patient with endometrial cancer is a considerable problem in gynecological oncology. Radical treatment is in opposition to the desire for maternity. All decisions should be made by the patient, and her point of view should be respected. However, she should be informed about all aspects. The quality of life is one of the most important targets, but it has to be stressed that radical treatment allows for a longer life without progression. Precision movements, less blood loss and shorter hospitalizations favor this surgical method in every aspect of the quality of life, especially for women of reproductive age, for whom diagnosis of cancer has a stronger impact on their lives.

## Data Availability

No new data were created or analyzed in this study.
